# The role of character positional frequency on Chinese word learning during natural reading

**DOI:** 10.1371/journal.pone.0187656

**Published:** 2017-11-14

**Authors:** Feifei Liang, Hazel I. Blythe, Xuejun Bai, Guoli Yan, Xin Li, Chuanli Zang, Simon P. Liversedge

**Affiliations:** 1 Academy of Psychology and Behavior, Tianjin Normal University, Tianjin, China; 2 Psychology, University of Southampton, Southampton, United Kingdom; Hangzhou Normal University, CHINA

## Abstract

Readers’ eye movements were recorded to examine the role of character positional frequency on Chinese lexical acquisition during reading and its possible modulation by word spacing. In Experiment 1, three types of pseudowords were constructed based on each character’s positional frequency, providing congruent, incongruent, and no positional word segmentation information. Each pseudoword was embedded into two sets of sentences, for the learning and the test phases. In the learning phase, half the participants read sentences in word-spaced format, and half in unspaced format. In the test phase, all participants read sentences in unspaced format. The results showed an inhibitory effect of character positional frequency upon the efficiency of word learning when processing incongruent pseudowords both in the learning and test phase, and also showed facilitatory effect of word spacing in the learning phase, but not at test. Most importantly, these two characteristics exerted independent influences on word segmentation. In Experiment 2, three analogous types of pseudowords were created whilst controlling for orthographic neighborhood size. The results of the two experiments were consistent, except that the effect of character positional frequency was absent in the test phase in Experiment 2. We argue that the positional frequency of a word’s constituent characters may influence the character-to-word assignment in a process that likely incorporates both lexical segmentation and identification.

## Introduction

Unlike most alphabetic scripts, Chinese text is generally printed as a string of continuous characters, with no visually distinct interword spaces. There is, therefore, no clear visual information available for Chinese readers to determine where the word boundaries lie within a sentence. Recently, there has been a great deal of interest in how Chinese readers segment and identify words within sentence contexts[1–8]. There has, however, been less research focusing on how readers segment and acquire novel words when they are encountered during reading, though see [9–10]. In the present study, the aim was to examine which word segmentation cues Chinese readers use when learning novel words in sentence contexts.

In alphabetic languages, the word is typically considered to be the basic processing unit of reading [11]. Words are salient due to the presence of visual boundaries: spaces between words. As a clear visual segmentation cue, spacing has been shown to be vital for efficient word identification and eye movement control in sentence reading [12, 13]. For example, in English text, when the spaces between words are removed, reading speed is dramatically reduced, fixations durations are increased, saccades become shorter, and the initial fixation on the word is transferred from the word center to the word beginning [11, 13, 14–19]. In addition, a number of different linguistic cues are used for morpheme boundaries in alphabetic writing systems such as bigram troughs in English [20], vowel harmony in Finnish [21–25], and the positional probability of characters within words in Thai [26–27].

To recapitulate, both visual and linguistic cues are used by readers to identify boundaries (for words, and for morphemes within words) in alphabetic writing systems. However, written Chinese, which is considered to be a largely logographic language, is extremely different from alphabetic writing systems. While there are many unique characteristics of the Chinese writing system [28, 29], we focus here upon those aspects that relate to word segmentation. First, written Chinese does not have spaces printed between words, most words consisting of between one and four adjacent characters, thus, there is no clear visual demarcation of word boundaries within a sentence. Second, when explicitly tested, there is often ambiguity with respect to the location of word boundaries in a sentence, both between and within individual Chinese readers [29, 30]. Third, the majority of Chinese characters (about 80%) can occur in all positions within a word (beginning, internal, or end positions). Fewer than 20% of Chinese characters unambiguously mark word boundaries (2.1% occurring solely as single-character words, 8.2% occurring solely at word beginnings, and 7.5% occurring solely at word endings) [31]. Furthermore, there is often temporary ambiguity concerning the segmentation of adjacent characters into words as a reader progresses through a sentence. For example, in the four-character string C1234 (专科学生, college student), it may be the case that C12 (专科, college), C23 (科学, science), and C34 (学生, student) are all words, but the only segmentation that fits the sentence context is C12 (专科, college) and C34 (学生, student). In such cases it seems likely, therefore, that higher-level processing of the sentence context (both semantic and syntactic) will be a vital aspect of word segmentation during reading, in order for that segmentation process to result in an accurate understanding of the sentence.

For the present experiment, a key theoretical question was considered in the context of these distinctive characteristics of written Chinese: which word segmentation cues do Chinese readers use when reading? To address this question, we examined learning of pseudowords embedded in sentences, allowing us to determine which of our manipulated segmentation cues impacted upon readers’ ability to identify and group together the constituent characters. The exploration of this question will inform and encourage developments in computational models of sentence and text-level reading that include non-alphabetic languages [32]. There are a number of computational models that can account for many of the behavioral phenomena associated with reading; however, most of those models have been developed to account for reading of alphabetic writing systems, such as E-Z Reader model[33], SWIFT [34], and GLENMORE [35]. Very few models have been extended to delineate the processes associated with sentence-level reading in non-alphabetic languages [32]. To date, the only exception is the model reported by Rayner, Li, and Pollatsek [36]. In this model, they maintained the basic assumptions of the E-Z Reader model (word-based processing) [33, 37, 38], but made a number of amendments in order to simulate Chinese text reading. Critically, however, this model does not directly address the issue of interest for our present experiment—the mechanism(s) by which word segmentation occurs in Chinese sentence reading. The purpose of the present study is to investigate which segmentation cues Chinese readers adopt in order to determine the locations of word boundaries and to identify those words within sentence contexts. There are a number of published studies which have investigated effects of such cues for word segmentation in Chinese.

One of the cues that might be used in lexical segmentation and identification is within-word positional information. In Chinese, some characters occur most frequently at the beginning of words (e.g., the frequency of the character “胡” at a word beginning is 47, whilst its frequency at a word end is 1, out of a total of 45,872 two-character words in the corpus of SUBTLEX-CH, 2010) [39], while other characters occur most frequently at the end of words (e.g., the frequency of the character “员” at a word beginning is 1, whilst its frequency at a word end is 46). Such statistical tendencies provide a cue to the reader as to the likely locations of word boundaries. Yen et al [6] investigated whether positional probability was used as a word boundary cue in lexical identification by Chinese readers; specifically, they examined whether or not a reader’s processing of overlapping candidate words was modulated by the constituent characters’ positional probabilities. The target words were existing words, with two characters long (C12), and the positional probability of the second character (C2) was manipulated. The results showed that readers had longer gaze durations and made more refixations when the positional frequency of the character was incongruent with its position within the target word compared to when its positional frequency was congruent with its position within the target word. It was argued that the probability of within-word character positions is a linguistic cue that Chinese adult readers use for word segmentation during the identification of lexical representations when reading. To the best of our knowledge, there have been no studies to address the issue in terms of the role of character positional frequencies in lexical acquisition during Chinese reading. Thus, the first purpose of the present study is to examine whether Chinese readers use these positional frequencies as word segmentation cues to identify the novel lexical representations within contexts during natural reading.

In addition, a recent study reported by Blythe et al [10] showed that inserting spaces between words facilitated the reading and learning of novel words within sentential contexts both for native Chinese children and adults although written Chinese has no spaces between words. In particular, the benefit of inter-word spacing to children’s reading of new vocabulary was maintained over time to subsequent encounters, where the newly acquired target words were then presented in a traditional, unspaced format. Skilled adult readers benefited from inter-word spacing when the new vocabulary was first encountered, but this benefit was relatively short-lived; once the words had been encountered four times then all adults appeared to read those words quickly and easily irrespective of whether they had been learned in a spaced or an unspaced format. Thus, these data suggest that once a word has been instantiated in a skilled adult reader’s lexicon then there is relatively little benefit to be gained from clear visual demarcation of word boundaries.

These data support the findings of Bai et al.’s study [1]: inserting inter-word spaces neither facilitated nor hindered the reading of Chinese text for native Chinese adults. Thus, it seems clear that when the content of the printed text is familiar to the reader (e.g., all words are known), then the presence of visual word boundaries is not particularly beneficial. The different patterns of results obtained for spacing effects for normal lexical identification, as opposed to lexical acquisition during sentence reading, can be explained within Li et al.’s model of Chinese word segmentation and word recognition [3]. Li et al. argued that word segmentation is an interactive process based on both top-down processing (activation from existing word representations in the mental lexicon) and bottom-up processing (activation from character-level representations on the basis of the fixated orthographic input). For a reader’s first encounter with a novel word, there is no top-down, word-level information to feed back into the process of word segmentation. Thus, bottom-up processing on the basis of the orthographic input seems to dominate the segmentation of text containing an unfamiliar word. In such a case, providing visual cues that clearly demark where the word boundaries are reduces the requirement for the reader to segment the text on the basis of the sentential context. Spaces between words can, therefore, facilitate reading of novel words. More commonly, when there are no unknown words in the text, existing representations directly boost the activation of a word’s constituent characters via top-down processing in order to facilitate activation of that word in combination with the bottom-up orthographic input. In this case, according to the model, it seems likely that visual cues to mark word boundaries will have a less important role to play in the segmentation of sentences containing familiar words.

In summary, in the case of learning new words embedded in sentences, research has indicated that although there is no visual word segmentation cue (i.e., word spacing) for Chinese readers to use in normal printed text, the artificial insertion of spaces between words has been shown to be an effective manipulation that helps readers to segment and identify novel words within sentence contexts [10]. As noted earlier, character positional probability has been shown to be one kind of linguistic cue that might be used by Chinese readers to facilitate the process of lexical acquisition. The second purpose of the present study was, therefore, to examine whether providing a visual word segmentation cue (i.e., word spacing) would facilitate Chinese readers’ use of linguistic cues (i.e., character positional frequencies) in lexical acquisition.

With respect to the manipulation of positional character frequencies, three types of two character pseudowords were constructed, based on the probability of within-word positions for each constituent character. Here, in the congruent condition, the position of each character was consistent with its most frequent within-word position; therefore, the positional frequencies of the constituent characters provided accurate information about the likely locations of each pseudoword’s boundaries. Second, in the incongruent condition, the position of each character conflicted with its most frequent within-word position, thus providing incorrect information about the likely locations of word boundaries. Third, in the balanced condition, characters were selected that had approximately 50% probability of occurring at the beginning or end of words; the positional frequencies in this condition did not provide a cue as to the likely location of the target words’ boundaries. Every pseudoword was embedded into six sentences, each of which was constructed to provide a meaningful context that would help readers to form an understanding of the new vocabulary. Half the sentences for each target word were read in a learning phase, where participants were told that they would encounter unknown vocabulary and were instructed to try and understand the meaning of the sentences. In the learning phase, a second word segmentation cue was also manipulated—word spacing. Half the participants read traditional, unspaced text while the other half of participants read word-spaced text. The second group of sentences for each target word was read in a test phase in which all participants read unspaced text. In addition, participants were required to make a semantic category judgment about each new word in the test phase.

We made two predictions relating to the basic manipulations within this experiment. First, with respect to the manipulation of the constituent characters’ positional frequencies, we predicted a similar pattern to that reported by Yen et al [6]. Specifically, we predicted that reading times on the target words would be shortest in the congruent condition (reflecting minimal processing difficulty) and longest in the incongruent condition, with reading times in the balanced condition falling in between. Such a pattern would reflect participants’ ability to make use of the constituent characters’ positional frequencies as part of the process for determining the locations of boundaries for new vocabulary when encountered in a sentence context. Second, with respect to the spacing manipulation, we predicted that we would replicate the pattern reported by Blythe et al [10] in which skilled adult readers benefited from word spacing when initially encountering new vocabulary (in the learning phase), but this benefit was not maintained in the test phase. Those results were argued to show that a lexical representation had been formed for each new word during the learning phase and thus, during subsequent encounters in the test phase skilled adult readers could process the new words quickly and easily, irrespective of the format in which they had been learned.

We also predicted an interaction between our manipulations of word spacing and positional frequencies in the learning phase, given our argument that both manipulations should affect the process of lexical acquisition by demarking word boundaries. When word spacing is present, this should to some extent override the need for lexical segmentation on the basis of linguistic cues, and so the effect of the positional frequency manipulation should be attenuated in the word spaced condition compared to the unspaced condition. With respect to the data from the test phase, we predicted an effect of the positional frequency manipulation again here—pseudowords in the congruent condition should be orthographically more familiar than pseudowords in the incongruent condition, and we predicted that this should impact upon the ease of lexical identification (again, with shorter reading times for the congruent pseudowords and longer reading times for the incongruent pseudowords). Finally, given that we did not expect our spacing manipulation in the learning phase would maintain to the subsequent test phase, we did not predict an interaction in the test phase between these two experimental manipulations (in contrast to our predicted interaction in the learning phase).

## Experiment 1

### Method

#### Ethics statement

The study was approved by the Institutional Review Board of the Academy of Psychology and Behavior, Tianjin Normal University. All participants provided written, informed consent before taking part in our experiments.

#### Participants

Sixty undergraduates (24 males and 36 females) from Tianjin Normal University were paid to take part in the eye tracking experiment, with a mean age of 22.1 years (range = 19- to 24-years). They were native Chinese speakers, with normal or corrected-to-normal vision. All of them were right-handed, and naïve regarding the purpose of the present study.

#### Materials and design

We calculated the probability of each character’s within-word position by its type frequency (data were from the corpus of SUBTLEX-CH) [39]. It was defined as the number of two-character words which contained that character in the beginning or end position, divided by the total number of two-character words which contained that character. Three groups of twenty characters were selected: (1) characters that had a high word-beginning probability (mean = 0.94; range = 0.90 to 0.98), and where the total number of two-character words containing that character in the first position (mean = 54) was at least eight times greater than the total number of two-character words containing that character in the final position (mean = 4); (2) characters that had a high word-end probability (mean 0.95; range = 0.90 to 1.00), and where the total number of two-character words containing that character in the final position (mean = 75) was at least ten times greater than the total number of two-character words containing that character in the first position (mean = 5); (3) characters that had close to equal word-beginning and -end probabilities (beginning mean = 0.50, range = 0.46 to 0.53; end mean = 0.50, range = 0.47 to 0.54). The number of strokes per character (range = 3 to 10), and character frequency (higher than 100 per million), were matched (see Table 1), so that there were no significant differences between these three groups of selected characters (both *F*s < 2.0, both *p*s > 0.05). We also calculated the mean token frequency of each character, and no significant differences were found across three groups of selected characters (*F*(2, 58) = 0.89, *p* > 0.05)

**Table 1:** Example and properties of the selected characters in Experiment 1.

	Character type		
	Word beginning	Word ending	Both
Example	挑(select)	尔(so)	子(son)
Pinyin	tiao1	er3	zi3
Mean character token frequency (per million)	529	539	520
Mean numbers of strokes	6.7	7.1	6.9

Using these selected groups of characters, three types of two-character pseudowords were constructed: (1) congruent pseudowords were comprised of a character with high word-beginning frequency as C1 and a character with high word-end frequency as C2; (2) incongruent pseudowords were comprised of a character with high word-end frequency as C1 and a character with high word-beginning frequency as C2; (3) balanced pseudowords were comprised of two characters with balanced word-beginning and word-end characters as C1 and C2. Pseudowords, as opposed to real words, were selected as target words for this experiment for two reasons. First, there are not enough real two-character words that satisfy our criteria regarding within-word positional probabilities for both constituent characters. Second, using pseudowords allowed us to ensure that all the target words were entirely unknown by skilled adult readers; this was vital given that our experimental task focuses on the learning of new vocabulary. Note that the behavior associated with learning unknown (but real) words, and that associated with learning pseudowords has been shown to be highly similar. There is, therefore, no reason to believe that the use of pseudowords would elicit atypical cognitive processing compared to what might occur more normally when encountering and learning new vocabulary during reading.

To ensure that the pseudowords were genuinely novel, and would not be mistakenly associated with real words, we took two precautions. First, we ensured that these pseudowords did not appear in the Contemporary Chinese Dictionary [40]. Second, in order to make sure the pinyin (the phonological information associated with a Chinese character) of each pseudoword did not resemble that of a real word, 15 participants who did not take part in the main eye tracking experiment were presented with a list of the *pinyin* for the 40 candidate pseudowords as well as 20 filler words (written using the Roman alphabet). They were instructed to write down pairs of characters that they thought were consistent with the *pinyin* and that formed real words. We selected 30 pseudowords for which all participants reported that they could not think of any real words that could possibly result from the *pinyins* for the two constituent characters; ten congruent pseudowords, ten incongruent pseudowords, and ten balanced pseudowords. Each constituent character only appeared once in a single target word. Each of the final set of pseudowords was designated as a novel (non-existent) item in one of ten real-world semantic categories (*e*.*g*., “挑尔” was designated to be a type of fruit that does not exist in the real world). The categories were: fruit, animal, clothes, container, architecture, medicine, transport tools, game, career, and flower. Each of the ten categories included three pseudowords, one in each of the positional frequency conditions (*e*.*g*., the category of fruit contained three pseudowords: “挑尔” in the congruent condition, “子左” in the incongruent condition, and “皮合” in the balanced condition).

There were two phases in the experiment: the learning phase and the test phase. In the learning phase, there were two presentation conditions: half the participants read the sentences presented as traditional, unspaced text while the other half read the sentences presented in a word-spaced format. In the test phase, all the participants read sentences presented in an unspaced format. For every semantic category (containing three pseudowords) three sets of six sentence frames were constructed, each of which provided a context describing that item (see Table 2 for examples). Three of each set of sentences were used in the learning phase, and the other three of each set were used in the test phase. Within each phase of the experiment, three sentences that contained the same target word were bound together, and presented one by one. Every participant read every pseudoword, but the sentence frames were counterbalanced between participants (within the sets of three associated with each target word) so that they only read each sentence frame once. This resulted in three counterbalanced lists of stimuli in total; each participant read one list of 180 experimental sentences associated with thirty pseudowords, consisting of thirty sets of three learning phase sentences and thirty sets of three test phase sentences. In both the learning and test phases, one of the three sentences per target word was followed by a yes/no comprehension question to ensure that participants were reading for meaning, and were able to understand the sentences. Further, in order to check that readers were able to learn the pseudowords from the sentences that we created, we included a semantic category judgment for each target word in the test phase of the experiment. For each pseudoword, we prepared a list of five semantic categories that were being used in the experiment, including the correct category for that pseudoword, as well as five distractor categories (i.e., weather, furniture, diploma, book and color). After reading the sentences associated with each pseudoword, participants were presented with the list of ten semantic categories and were asked to decide which category that pseudoword belonged to. In total, each participant read 93 sentences (the first 3 sentences were practice items), and responded to 31 comprehension questions in each experimental phase. In addition, each participant made 31 semantic category judgments in the test phase as well.

**Table 2:** Illustration of the example and the procedure of the experiment.

Experiment phase	Sentence frame	Sentences with unspaced and spaced presentation conditions and translations
Learning phase	1	Unpsaced: 盛产于非洲的挑尔/子左/皮合味道很特别。
		Spaced: 盛产 于 非洲 的 挑尔/子左/皮合 味道 很 特别。
		Translation: ***Tiaoer/zizuo/pihe***, with special taste, is abundant in Africa.
	2	Unspaced: 两个西瓜大约有一个挑尔/子左/皮合那么大。
		Spaced: 两个 西瓜 大约 有 一个 挑尔/子左/皮合 那么 大。
		Translation: The ***tiaoer/zizuo/pihe*** is as the same size as two melons.
	3	Unspaced: 米黄色的挑尔/子左/皮合全部倒挂在树枝上。
		Spaced: 米黄色 的 挑尔/子左/皮合 全部 倒挂 在 树枝 上。
		Translation: The yellow ***tiaoer/zizuo/pihe*** hangs upside down from the branches of the tree.
Test phase	4	Unspaced: 没有熟透的挑尔/子左/皮合是绝对不能吃的。
		Translation: Unripe ***tiaoer/zizuo/pihe*** definitely should not be eaten.
	5	Unspaced: 一个挑尔/子左/皮合居然比一公斤牛肉都贵。
		Translation: The price of each ***tiaoer/zizuo/pihe*** is higher than that of one kilogram of beef.
	6	Unspaced: 剥去外皮就能看见挑尔/子左/皮合的果肉。
		Translation: The skin of the ***tiaoer/zizuo/pihe*** is peeled, and then the flesh is seen.
Multiple choice question		请问: 挑尔/子左/皮合属于以下哪个类别?
		(Question: Which category does *tiaoer/zizuo/pihe* belong to?)
		职业(career) 动物(animal) 水果(fruit) 书本(book) 交通工具(transportation tools)
		电器(electric equipment) 家具(furniture) 酒类(wine) 衣服(clothes) 颜色(color)

Note that, the three two-character pseudowords for each sentence frame are shown as an example in order to demonstrate our counterbalancing procedure. Only one pseudoword was presented within each sentence in the formal eye movement experiment.

The target words never appeared as the first or final word of a sentence. The mean length of the sentences was 13.5 characters (range = 13 to 14 characters) which, on average, consisted of 8.0 words (range = 6 to 10 words). The naturalness and the difficulty of the sentences were rated by two different groups of 15 undergraduates from Tianjin Normal University who did not take part in the main eye tracking experiment, using a 5-point scale. A score of “1” meant that the sentence was deemed very unnatural/ very difficult to understand, and a score of “5” meant that the sentence was deemed very natural/ very easy to understand). Before carrying out these assessments of the stimuli, we replaced the pseudowords in the sentences (which were obviously meaningless) with real words which belonged to the same semantic category as the pseudoword. This allowed us to evaluate how reasonable the sentences were to read independent of any oddity arising due to the inclusion of the pseudoword. The mean rating for naturalness was 4.46 (SD = 0.16) and the mean rating for difficulty was 4.20 (SD = 0.46), indicating that the sentences constructed for the experiment were very natural and easy for participants to read. Importantly, we also pre-screened these sentences for the extent to which different readers would agree on the locations of word boundaries. Another 15 undergraduates who did not take part in the main eye tracking experiment were presented with the sentences written in a word-spaced format, and were required to judge whether or not they agreed with the experimenters’ word segmentation (the pseudowords were clearly denoted within the sentence, and participants were instructed to ignore those). If they did not agree with the experimenter’s segmentation, they were required to mark what they considered to be the correct word boundary. Agreement on this task was calculated as per Yan et al [41]. Participants were very consistent with the experimenter’s word segmentation, with a mean agreement of 98.9% (SD = 1.3%; range = 94% to 100%).

Finally, we calculated the probability of the target characters combining with those from adjacent words to form plausible pseudowords. There were no significant differences across conditions, *F* (2, 179) = 0.78, *p* > 0.05.

#### Apparatus

Participants’ eye movements were recorded with a SR Research EyeLink 1000 eye tracker (sampling rate = 1000Hz) that monitored the position of the right eye (viewing was binocular). Sentences were presented on a 19-inch DELL monitor with a 1024 × 768 pixel resolution. Sentences were presented in black, Song font size 18, and each character extended 25× 25 pixels (with a space of 1 pixel between characters in the unspaced condition). Participants were seated 75 cm from the video monitor; at this distance, one character subtended 0.74° visual angle.

#### Procedure

Participants were instructed to lean on chin and forehead rests in order to minimize head movements. Prior to the start of the experiment, there was a calibration procedure (the maximum calibration error accepted was 0.2 deg). Once the eye tracker had been calibrated, the sentences were presented one at a time. The calibration was checked after each sentence, and recalibration was carried out during the experiment whenever necessary.

In the learning phase, participants were instructed to read the sentences normally and carefully. They were informed that the sentences would contain some words that they did not know, and that they should try to understand the sentences as best as they could. Participants were asked to click the mouse to end the display when they had finished reading each sentence. Then, they were asked to use the mouse to click within pre-defined regions on the screen corresponding to the answers for the questions (for the comprehension questions, there were two different regions representing “yes” and “no” responses; for the semantic category judgments there were ten regions representing the ten categories). The learning phase lasted approximately 40 minutes. Participants were then asked to leave the lab and to return after one hour for the test phase. In the test phase, they were instructed to read the sentences normally, and to answer the comprehension questions and the multiple choice questions using the mouse. They were not informed prior to the test phase that the sentences they would read were related to the new words they had encountered in the learning phase. In total, the test phase lasted about 60 minutes.

### Results

The overall accuracy score for the comprehension questions was 86.6% in the learning phase and 95.3% in the test phase, indicating that all the participants read and understood the meaning of the sentences in both phases. The slightly lower accuracy score in the learning phase was most likely a consequence of the fact that the learning phase contained the 1^st^-4^th^ encounter with each target word, compared to the 5^th^-8^th^ encounters with each target word in the test phase (3 times in different sentence frames and 1 time in the comprehension question in each phase). The mean score for the semantic category judgments was 93.2%, demonstrating that readers were able to classify each pseudoword into the correct semantic category during the test phase.

The data set was trimmed on the basis of three criteria, as per [1, 4, 10]: (1) trials on which tracker loss occurred were excluded from the analyses; (2) trials in which fewer than four fixations were made in total were excluded; and (3) fixations shorter than 80 ms or longer than 1200 ms were removed. In total, 1.8% of the data were excluded from the analysis.

We report local analyses of eye movement data from the target words, focusing on three key reading time measures: (1) first fixation duration, the duration of the initial, first pass fixation on the target word regardless of how many fixations were made in total; (2) gaze duration, the sum of all first pass fixation durations on the target word before the eyes moved to another word in the sentence; and (3) total fixation time, the sum of all fixation durations on the target word. We also analyzed three other eye movement measures—the probability of refixation, the probability of regressions back from the target word, and total number of fixations. The patterns based on these data sets were highly consistent with those for total fixation time in both two experiments. For brevity, we only reported the results of three critical reading time measures. The means and standard deviations for the eye movement measures are shown in Table 3.

**Table 3:** The mean and the standard deviation of all the eye movement measures in all conditions in Experiment 1.

Measures	Pseudoword type	Learning		Test	
		Unspaced	Spaced	Unspaced	Spaced
First fixation duration (ms)	Congruent	272(102)	272(109)	262(95)	267(103)
	Balance	272(105)	284(113)	263(98)	275(101)
	Incongruent	289(120)	283(116)	274(104)	284(108)
Gaze duration (ms)	Congruent	375(208)	343(186)	329(163)	337(177)
	Balance	380(195)	353(193)	344(187)	353(183)
	Incongruent	410(226)	375(213)	361(202)	368(194)
Total fixation time (ms)	Congruent	683(464)	600(412)	550(360)	623(396)
	Balance	682(432)	612(415)	576(385)	616(402)
	Incongruent	764(482)	668(475)	632(419)	652(419)

For the analyses we conducted linear-mixed-effects models using the lme4 package (version 1.1–7) in R [42]. As fixed factors we included *Pseudoword Type*, *Experiment Phase*, *Learning group* and their interactions. A random model including intercepts and possible slopes for the main effects and their interactions with participants and items as random factors did not converge for the dependent measures [43]. Thus, we ran a model with intercepts for the main effects with participants as a random factor and with intercepts for the items as random factors. The fixation times were analyzed using log-transformed data to reduce distribution skewing [44]. Planned contrasts were carried out on all dependent measures to examine the positional frequency effect on the basis of our hypothesis: the first contrast compared the congruent and balanced condition, and the second contrast compared the congruent and incongruent condition. For each contrast we report regression coefficient (b), standard error (SE), and t statistics for reading time data. T-values greater than 1.96 were considered significant at the 5% level. Fixed effect estimations for the fixation times are shown in Table 4, and other planned contrasts to further investigate the differences between experimental conditions are reported in Table 5.

**Table 4:** Fixed effect estimates for the first fixation duration, gaze duration and total fixation time on the target pseudowords in Experiment 1.

	First fixation duration			Gaze duration			Total fixation time		
	*b*	*SE*	*t*	*b*	*SE*	*t*	*b*	*SE*	*t*
Interpret	5.54	0.01	385.18***	5.75	0.02	291.68***	6.24	0.03	200.32***
Pseudoword Type 1^a^	-0.02	0.01	-1.44	-0.03	0.03	-1.15	-0.01	0.04	-0.19
Pseudoword Type 2^b^	0.04	0.01	3.21**	0.08	0.03	2.87**	0.09	0.04	2.18*
Learning Group	-0.01	0.03	-0.60	0.03	0.03	0.90	0.03	0.05	0.63
Experiment Phase	-0.02	0.01	-2.56*	-0.05	0.01	-5.56***	-0.09	0.01	-6.98***
Pseudoword Type 1^a^ × Learning Group	0.04	0.02	2.11*	0.01	0.02	0.61	-0.01	0.03	-0.30
Pseudoword Type 2^b^ × Learning Group	-0.01	0.02	-0.34	-0.01	0.02	-0.33	0.05	0.03	1.50
Pseudoword Type 1^a^× Experiment Phase	-0.002	0.02	-0.11	-0.01	0.02	-0.68	0.00	0.03	0.004
Pseudoword Type 2^b^ × Experiment Phase	0.01	0.02	0.61	0.001	0.02	0.04	-0.02	0.03	-0.56
Learning Group× Experiment Phase	-0.03	0.01	-1.80^§^	-0.11	0.02	-5.57***	-0.23	0.03	-9.12***
Pseudoword Type 1^a^ × Learning Group× Experiment Phase	-0.01	0.04	-0.26	0.01	0.05	0.28	-0.05	0.06	-0.88
Pseudoword Type 2^b^ × Learning Group× Experiment Phase	-0.03	0.04	-0.83	-0.02	0.05	-0.31	0.04	0.06	0.64

^a^ Refers to the comparison between the balanced and congruent condition;

^b^ Refers to the comparison between the congruent and incongruent condition.

*** p < .001;

** p < .01;

* p < .05;

^§^ p < .10

**Table 5:** Planned contrasts from the main models reported in Table 3.

	*b*	*SE*	*t*
First fixation duration			
Contrast 1^a^	-0.006	0.01	-0.55
Contrast 2^b^	-0.03	0.01	-3.10**
Contrast 3^c^	-0.04	0.02	-2.16*
Contrast 4^d^	-0.001	0.02	-0.07
Gaze duration			
Contrast 1^a^	-0.00	0.01	-0.006
Contrast 2^b^	-0.11	0.01	-7.91***
Total fixation time			
Contrast 1^a^	0.03	0.02	1.50
Contrast 2^b^	-0.20	0.02	-11.45***

^a^ Refers to the comparison between the learning and test phase for the spaced learning group;

^b^ Refers to the comparison between the learning and test phase for the unspaced learning group;

^c^ Refers to the comparison between the congruent and balanced condition for the spaced learning group;

^d^ Refers to the comparison between the congruent and balanced condition for the unspaced learning group.

Readers had longer reading time for pseudowords in the incongruent condition than those in the congruent condition (first fixation duration: mean difference = 15ms; gaze duration: mean difference = 43ms; total fixation time: mean difference = 66ms). In contrast, no reliable differences were found between the congruent and balanced pseudowords on all dependent measures. We also directly compared the incongruent and balanced conditions for all three eye movement measures. There was a tendency for readers to have longer reading times on pseudowords in the incongruent condition than pseudowords in the balanced condition; the effect was marginally significant for first fixation duration (mean difference = 9ms, *b* = -0.02, *SE* = 0.01, *t* = -1.78, *p* = 0.09) and gaze duration (mean difference = 21ms, *b* = -0.04, *SE* = 0.03, *t* = -1.73, *p* = 0.09), and significant for total fixation time (mean difference = 58ms, *b* = -0.08, *SE* = 0.04, *t* = -1.99, *p* < 0.05). These results showed that, when a pseudoword was made up of two constituent characters for which the within-word positions were incongruent with their usual positions within Chinese words, the readers experienced significant processing difficulty. The interactions between *Pseudoword Type* and *Experiment Phase* were not significant for any of the three reading time measures, indicating that the influence of the constituent characters’ positional frequencies on lexical processing occurred consistently in both the learning and test phases (e.g., across the multiple encounters with each pseudoword when embedded in different explanatory sentence frames).

There was a robust main effect of *Experiment Phase* on all three measures, showing that reading times on the pseudowords in the learning phase were longer than those in the test phase (first fixation duration: mean difference = 8ms; gaze duration: mean difference = 24ms; total fixation time: mean difference = 61ms). This effect replicates that obtained by Blythe et al.’s study [10] and suggests that as the reader experienced more encounters with each pseudoword in different sentence frames, reading time on that novel word gradually became shorter.

On first fixation duration, there was neither a reliable effect of *Learning Group*, nor an interaction between *Learning Group* and *Experiment Phase*. Thus, both the spaced and unspaced learning groups had similar first fixation times on the target pseudowords in both the learning and test phases. Similarly, the main effect of *Learning Group* was not reliable in either gaze duration or total fixation time. In contrast to the first fixation data, however, we did observe a robust interaction between *Learning group* and *Experiment phase* on these two later measures. We conducted two additional contrasts to further examine how the two groups of participants differed between the learning and test phases. Participants in the spaced learning group showed no significant differences in reading times between the learning and test phases, indicating that the presence of word spacing allowed them to process the pseudowords efficiently even during their initial encounters with those novel orthographic forms (learning phase mean gaze = 357 ms, test phase mean gaze = 353 ms; learning phase mean total time = 627 ms, test phase mean total time = 630 ms; Contrasts labeled 1 in Table 5). In contrast, participants in the unspaced learning group had significantly longer reading times on pseudowords in the learning phase as compared to the test phase (learning phase mean gaze = 389 ms; test phase mean gaze = 345 ms; learning phase mean total time = 710, test phase mean total time = 586 ms; Contrasts labeled 2 in Table 5). These results showed, therefore, that the unspaced learning group were at a disadvantage in processing the pseudowords in the learning phase when there were no visual segmentation cues, and that this group exhibited a significant reduction in reading times over the repeated encounters with these orthographic forms. The spaced learning group, however, were relatively efficient in processing the pseudowords even during the initial encounters in the learning phase.

When considering the interactions between *Pseudoword Type* and *Learning Group*, there was a small effect in first fixation such that first fixation durations on congruent pseudowords were, on average, 9 ms shorter than those on balanced pseudowords for the spaced learning group, but not for the unspaced learning group. We had no a prior rationale for this pattern, and the effect was extremely small in magnitude. Furthermore, there was no significant interaction in either gaze duration or total fixation time. Thus, counter to our predictions, the dominant pattern in these data indicates that the effects of both word spacing and of characters’ positional frequencies that we observed were independent in terms of their influence on lexical processing. We return to this point in the Discussion.

### Discussion

In Experiment 1, a pseudoword learning paradigm was used to examine the relative influence of two segmentation cues—the positional frequency of the word’s constituent characters, and the presence/ absence of word spacing—on Chinese word learning. Specifically we examined whether word spacing would facilitate Chinese readers’ processing of linguistic cues for successful word segmentation when learning novel words within contexts.

First, the data showed clear differences between learning novel words in a traditional, unspaced format compared to a word-spaced format. Specifically, reading times on the pseudowords were relatively similar, and shorter, for readers in the spaced learning group in both the learning and test phases. In contrast, readers in the unspaced learning group were at a disadvantage in the learning phase and their reading times decreased significantly in the test phase. The present results replicated the previous findings [10], indicating that the insertion of word spacing, visually demarking the word boundaries, helped readers to segment and identify the novel words within the initial several contexts more quickly, and to form new lexical representations of those words. In contrast, readers in the unspaced learning group processed the novel word less efficiently during the initial encounters of the learning phase due to the absence of visual word boundary cues. It was only in the test phase, after numerous exposures to each pseudoword, that readers in the unspaced learning group were able to process them as efficiently as their peers who had been provided with visual segmentation cues in the learning phase.

As predicted, we also observed significant influences of our manipulation of the constituent characters’ positional frequencies. Participants had longer reading times on pseudowords for which the positional frequency cues were incongruent compared to those pseudowords in which the cues were either congruent or were balanced (e.g., did not provide a strong cue as to within-word location). This pattern was consistent on all eye movement measures. The influence of the positional frequencies of characters has been previously observed on reading times for known words [6], and here we demonstrate that this cue is also important with respect to the learning of novel words. Let us now consider three additional, important points relating to the effect of positional frequencies.

First, we found no interaction of character positional frequency with experiment phase, which means that the character position effect occurred in both the learning and test phases. This result indicates that positional character frequency influences both the ease with which a new representation is instantiated in the lexicon, as well as the ease with which such a representation is subsequently accessed in the lexicon after that representation has been instantiated.

Second, it is clear that the effect of the positional frequency manipulation was mostly due to disruption from the incongruent condition. Contrary to our hypotheses, there were no significant differences between the balanced and congruent conditions. There was one aspect of the experimental stimuli that we considered might account for this pattern of effects—position-specific neighborhood size. Previous work has shown that processing times for words with more position-specific neighbors were shorter than those words with fewer position-specific neighbors when recognizing Chinese words, both in a lexical decision task and natural sentence reading [45]. With respect to the manipulation of positional frequencies in the present study, by virtue of the criteria by which these pseudowords were selected, it seems likely that this manipulation is inherently linked to position-specific neighborhood size. For example, if a pseudoword’s initial character is extremely common as the first character of Chinese words then, by definition, it will have a high number of orthographic neighbors. The congruent and incongruent pseudowords created for the present experiment were selected on the basis that both of their constituent characters were strongly biased in terms of their within-word positional probability. We calculated position-specific neighborhood size for each of the three types of pseudoword (congruent, balanced, and incongruent), as the summation of the number of real two-character words containing the first character in that position and the number of real two character-words containing the final character in that position (using the SUBTLEX-CH corpus) [39]. For example, the number of position-specific neighbors of the target word “挑尔” were 15(the frequency of the first character “挑” at word beginning) plus 147(the frequency of the second character “尔” at word ending), which equalled 162. An analysis showed that the neighborhood sizes for both congruent (M = 86.1) and balanced pseudowords (M = 92.9) were significantly larger than those for incongruent pseudowords (M = 11.8, both *t*s > 3, both *p*s < 0.01), but there was no difference between the balanced and congruent conditions (*t*(18) = 0.29, *p* > 0.05). Recall that, within the data from the present experiment, the effect of positional frequencies was driven by increased processing difficulty for the incongruent pseudowords and there were no significant differences between congruent and balanced pseudowords. Given the overlap in the pattern of differences between the conditions, therefore, it seems feasible that differences in neighborhood size might underlie the effects associated with our manipulation of positional frequencies. It should be noted here that the position-specific neighborhood size was not explicitly manipulated in the present experiment. The first goal of Experiment 2 was, therefore, to disentangle the influences of these variables by manipulating positional frequencies whilst controlling position-specific neighborhood size.

Third, and somewhat surprisingly, we found no clear evidence for an interaction between character positional frequency and the spacing manipulation. We had considered it likely that the presence of spacing, a very salient visual cue to word segmentation, would override any effects of positional frequency due to the reduced need for the reader to process that information when determining a new word’s constituent characters. In contrast to this hypothesis, the results showed a consistent degree of disruption from incongruent positional frequencies, irrespective of the presence or absence of word spacing. It appears that the positional frequency of characters has a strong influence on the ease with which readers process novel pseudowords regardless of how the text is visually presented (see also Liang et al., 2015). This pattern in the data from Experiment 1 led us to consider exactly how our manipulation of characters’ positional frequencies might be affecting lexical segmentation in Chinese reading.

Li et al. argued that there are two possibilities with respect to Chinese lexical segmentation—the feed-forward hypothesis, and the holistic hypothesis [3]. In the feed-forward hypothesis, three stages are proposed—character recognition, then word segmentation, and then lexical identification; it is only this final, lexical identification process that is influenced by top-down information from word-level representations. In the holistic hypothesis, as implemented in Li et al.’s model [3], word segmentation and lexical identification are not distinct from each other. Furthermore, word-level representations can feed back, providing top-down influence to the character recognition level–*“If a character is a part of a word with high activation*, *it will receive more evidence from the word recognition level”* (pp. 545). As discussed earlier, one aspect of lexical representations that is known to influence to ease of lexical processing is orthographic neighborhood size. Whilst this factor was not controlled in Experiment 1, it was in Experiment 2. The second goal of Experiment 2 was, therefore, to investigate whether our manipulation of character positional frequencies, when controlled for neighborhood size, was consistent with the feed-forward or the holistic hypothesis.

## Experiment 2

In Experiment 2, the same three types of pseudowords as in Experiment 1 were constructed based on each character’s positional frequency while the position-specific neighborhood size was controlled across these three conditions. First, and foremost, if an effect of character positional frequency was still found on reading times, then this would support our original premise that readers are sensitive to such statistical cues when learning new words through reading.

With respect to the second goal for Experiment 2, the feed-forward and holistic hypotheses predicted distinct patterns of results when overall neighborhood size was controlled. First, the feed-forward hypothesis would predict clear differences between all three positional frequency conditions, with the shortest reading times on congruent pseudowords and the longest reading times on incongruent pseudowords. The positional frequency cues should be associated with character-level representations, feeding directly into lexical segmentation with no top-down activation from word-level representations. For this reason, the characters that comprised the congruent pseudowords should give the reader strong, and accurate, cues about the locations of word boundaries whilst the characters comprising the incongruent pseudowords should give the reader strong cues that directly contradict the correct segmentation for that particular section of the text. The characters that make up the balanced pseudowords should neither help nor hinder lexical segmentation, since they do not contain any useful cues as to the location of word boundaries. Second, the holistic hypothesis predicts a quite different pattern of results from this manipulation. Since position-specific neighborhood size was controlled across conditions in Experiment 2, then all pseudowords should receive an equal amount of top-down activation from existing lexical entries that partially match both the identity and the within-word positions of the orthographic input. Thus, the holistic hypothesis would predict no differences in reading times between the three positional frequency conditions.

### Method

#### Participants

Another group of sixty undergraduates (28 males and 32 females) in Tianjin Normal University participated in Experiment 2, with a mean age of 22.4 years (range = 19- to 23-years). As in Experiment 1, they were all native speakers of Chinese with normal or corrected-to-normal vision, and they were all right-handed, and naïve regarding the purpose of the experiment.

#### Material & design

Three types of pseudowords were constructed as in Experiment 1, providing congruent, incongruent or balanced word segmentation cues, using three groups of characters: (1) characters with high word-beginning frequency (the probability of the characters occurring in the initial position of a word was 0.97, range = 0.93–1.00); (2) characters with high word-ending frequency (the probability of the characters occurring in the final position of a word was 0.95, range = 0.91–1.00); (3) characters with equal probability for occurring at the beginning or the end of a word (the probability of the characters occurring in the initial position of a word was 0.50, range = 0.44–0.56). As in Experiment 1, the number of strokes, and character’s token frequency in each group of selected characters were also matched (see Table 4; both *F*s < 1.8, both *p*s > 0.05). These three lists of characters were used to create the congruent, balanced, and incongruent 2-character pseudowords following the same procedure as reported for Experiment 1 (see Table 6).

**Table 6:** Example and properties of the selected characters in Experiment 2.

	Character type		
	Word beginning	Word ending	Both
Example	无	然	泪
Pinyin	wu1	ran2	lei4
Mean character frequency (per million)	231	581	198
Mean numbers of strokes	8.4	8.6	8.2

Overall neighborhood size was also controlled across these three types of pseudowords. This was calculated as the sum of the number of real, two-character words containing the pseudoword’s first character in that position, and the number of real two-character words containing the pseudoword’s final character in that position (using the SUBTLEX-CH corpus) [39]. The mean, position-specific neighborhood sizes for the congruent, balanced, and incongruent pseudowords were: 33.1, 29.3, 26.8, respectively, and there were no significant differences across the three experimental conditions (*F* (2, 27) = 1.4, *p* > 0.05). The sentence frames and design were identical to Experiment 1.

#### Apparatus & procedure

Both were identical to Experiment 1.

### Results

The mean score on the comprehension questions was 91.4% in the learning phase and 97.2% in the test phase indicating, again, that readers understood the sentences very well. The mean score on the semantic category judgments was 90.5% (SD = 7.3%), also suggesting that readers were able to learn the semantic categories of the pseudowords after six exposures in different sentence contexts.

The criteria for data trimming were identical to Experiment 1. In total, 1.7% of the data were removed prior to the conducting the statistical analyses. The analysis procedure was also identical to Experiment 1. Again, we report first fixation duration, gaze duration, and total fixation time for the target pseudoword in each sentence; means and standard deviations for each condition are shown in Table 7. Fixed effect estimates for the reading time analyses are shown in Table 8, and planned-contrasts were performed to further investigate the differences between experimental conditions. These are reported in Table 9.

**Table 7:** The mean and the standard deviation of all the eye movement measures in all conditions in Experiment 2.

Measures	Pseudoword type	Learning		Test	
		Unspaced	Spaced	Unspaced	Spaced
First fixation duration (ms)	Congruent	256(98)	250(99)	253(92)	254(93)
	Balance	264(100)	258(104)	259(100)	253(97)
	Incongruent	261(101)	270(112)	254(95)	255(98)
Gaze duration (ms)	Congruent	350(195)	332(184)	328(181)	330(177)
	Balance	359(195)	348(188)	331(178)	335(185)
	Incongruent	362(204)	362(197)	331(190)	346(196)
Total fixation time (ms)	Congruent	608(401)	567(394)	586(396)	569(359)
	Balance	618(395)	587(395)	578(382)	560(340)
	Incongruent	692(429)	621(385)	609(410)	608(392)

**Table 8:** Fixed effect estimates for the first fixation duration, gaze duration and total fixation time on the target pseudowords in Experiment 2.

	First fixation duration			Gaze duration			Total fixation time		
	*b*	*SE*	*t*	*b*	*SE*	*t*	*b*	*SE*	*t*
Interpret	5.48	0.01	390.3***	5.70	0.02	276.13***	6.18	0.03	197.15***
Pseudoword Type 1^a^	-0.02	0.01	-1.29	-0.03	0.02	-1.15	-0.02	0.03	-0.54
Pseudoword Type 2^b^	0.02	0.01	1.65	0.04	0.02	1.64	0.09	0.03	2.68*
Learning Group	0.01	0.03	0.34	-0.002	0.04	0.05	0.05	0.06	0.78
Experiment Phase	-0.01	0.01	-1.88^§^	-0.05	0.01	-5.13***	-0.05	0.01	-4.24***
Pseudoword Type 1^a^ × Learning Group	-0.02	0.02	-0.94	0.003	0.02	0.14	0.004	0.03	0.13
Pseudoword Type 2^b^ × Learning Group	-0.02	0.02	-1.31	-0.04	0.02	-1.65	0.007	0.03	0.21
Pseudoword Type 1^a^× Experiment Phase	0.03	0.02	1.56	0.04	0.02	1.63	0.06	0.03	1.82^§^
Pseudoword Type 2^b^ × Experiment Phase	-0.04	0.02	-2.37*	-0.05	0.02	-1.94^§^	-0.10	0.03	-3.36***
Learning Group× Experiment Phase	-0.004	0.01	-0.29	-0.04	0.02	-2.19*	-0.09	0.03	-3.38***
Pseudoword Type1^a^ × Learning Group× Experiment Phase	-0.02	0.04	-0.62	-0.02	0.05	-0.50	0.01	0.06	0.18
Pseudoword Type2^b^ × Learning Group× Experiment Phase	0.05	0.04	1.36	0.02	0.05	0.50	-0.06	0.06	-0.91

**Table 9:** Planned contrasts from the main models reported in Table 7.

		Contrast 1^a^	Contrast 2^b^	Contrast 3^c^	Contrast 4^d^
First fixation duration	*b*	-0.01	-0.02	0.04	0.001
	*SE*	0.01	0.01	0.02	0.02
	*t*	-1.13	-1.54	2.68**	0.05
Gaze duration	*b*	-0.03	-0.07	0.06	0.01
	*SE*	0.01	0.01	0.03	0.03
	*t*	-2.04*	-5.18***	2.35*	0.57
Total fixation time	*b*	-0.01	-0.10	0.14	0.04
	*SE*	0.02	0.02	0.04	0.04
	*t*	-0.60	-5.38***	3.84***	1.08

^a^ Refers to the comparison between the learning and test phase for the spaced learning group;

^b^ Refers to the comparison between the learning and test phase for the unspaced learning group;

^c^ Refers to the comparison between the congruent and incongruent condition in the learning phase;

^d^ Refers to the comparison between the congruent and incongruent condition in the test phase.

There were no significant differences between congruent and balanced pseudowords on all three eye movement measures (first fixation duration: mean difference = 5ms; gaze duration, mean difference = 8ms; total fixation time: mean difference = 4ms). Furthermore, the interaction between *Pseudoword Type* (congruent vs. balanced condition) and *Experiment Phase* was not reliable for first fixation duration, gaze duration, and total fixation time. These results replicated the pattern reported from Experiment 1, indicating that Chinese readers did not experience any increased processing difficulty when reading a balanced pseudoword (e.g., with no useful word boundary information) relative to when reading a congruent pseudoword (e.g., with useful word boundary information). The differences between congruent and incongruent pseudowords were not significant for either first fixation duration (mean difference = 7ms) or gaze duration (mean difference = 15ms) but were, significant for total fixation time (mean difference = 51ms). As in Experiment 1, we directly compared the incongruent and balanced conditions; there were no significant differences for first fixation duration (mean difference = 1ms, *b* = -0.001, *SE* = 0.01, *t* = -0.35, *p* > 0.05) or gaze duration (mean difference = 7ms, *b* = -0.01, *SE* = 0.02, *t* = -0.49, *p* > 0.05), but there was a significant difference for total fixation time (mean difference = 47ms, *b* = -0.07, *SE* = 0.03, *t* = -2.15, *p* < 0.05). These results shows that the disruptive effect associated with the incongruent pseudowords was observed only in later eye movement measures when the stimuli’s position-specific neighborhood size was controlled.

In contrast to the results from Experiment 1, the interaction between *Pseudoword Type* (congruent vs. incongruent conditions) and *Experiment Phase* was significant for first fixation duration and total reading time, and marginally significant for gaze duration (*p* = 0.05). The data showed that participants had shorter reading times on congruent pseudowords than on incongruent pseudowords in the learning phase (first fixation duration: mean difference = 13ms; gaze duration: mean difference = 21ms; total fixation time: mean difference = 69ms) but not in the test phase (first fixation duration: mean difference = 1ms; gaze duration: mean difference = 9ms; total fixation time: mean difference = 31ms). Taken together, these results suggest that when overall neighborhood size was controlled, the processing associated with incongruent pseudowords was delayed, but this delay was limited to the learning phase of the experiment (the positional frequency effect occurred both in the learning and test phase in Experiment 1).

The main effect of Experiment Phase was marginal for first fixation duration (*p* = 0.06), and significant for both gaze duration and total fixation time. Again, these results replicate the pattern of results from Experiment 1, showing that readers had longer reading times in the learning phase than in the test phase.

Although the main effect of *Learning Group* was not reliable in any of the dependent measures, there was a significant interaction between *Learning Group* and *Experiment Phase* for both gaze duration and total fixation time. As in Experiment 1, additional contrasts were run to explore this interaction further: Contrast 1 compared data from the learning and test phases for the spaced learning group, and Contrast 2 compared data from the learning and test phases for the unspaced learning group (see Table 9). The results showed that gaze durations on the pseudowords in the learning phase were longer than those in the test phase both for the spaced learning group (learning phase mean = 347ms; test phase mean = 337ms) and the unspaced learning group (learning phase mean = 357ms; test phase mean = 330ms). Again, however, the improvement for the unspaced learning group (27ms) was greater than that for the spaced learning group (10ms), replicating the pattern observed in Experiment 1. Finally, total fixation times on the pseudowords did not differ between the learning (M = 592ms) and test phase (M = 578ms) for readers in the spaced learning group, whilst there was a significant reduction in total fixation time between the learning (M = 639ms) and test (M = 590ms) phases for readers in the unspaced group. These results were consistent with the data from Experiment 1 showing that, due to the presence of word spacing as a segmentation cue, participants in the spaced learning group had relatively efficient reading times in the learning phase whilst there was a significant cost there for participants in the unspaced group.

### Discussion

In Experiment 2, we again found a disruptive effect associated with reading pseudowords with incongruent character positional frequencies, even after controlling for position-specific neighborhood size. Together with the data from Experiment 1, these results indicate that the statistical cues of characters’ positional frequencies, independent of neighborhood size, do influence Chinese word identification during reading.

With respect to the aspect of lexical identification that is influenced by characters’ positional frequencies, two contrasting predictions were made. In the case of a multi-stage process, whereby Chinese lexical segmentation is separate from, and precedes, lexical identification, significant differences were predicted between all three conditions of the manipulation (congruent, balanced, and incongruent). Alternatively, in the case of a single-stage process, whereby lexical segmentation and identification are combined and receive top-down information from word-level representations, then no differences were predicted between the three conditions (due to the fact that they were matched on neighborhood size, thus controlling for the degree of top-down activation). Clearly, the results did not match either of these two predictions entirely.

These data clearly contradict the notion of a multi-stage process in which lexical segmentation and identification are separate (as per the feed-forward hypothesis) [3]. In order to support such a model, the data would have to show both (a) facilitated processing from congruent positional frequencies relative to the baseline (balanced) condition; and (b) a cost to processing from incongruent positional frequencies relative to the baseline condition. The latter effect indicates that the incongruent cues may have hindered the process by which character representations were allocated to within-word positions. There was no evidence, however, for any facilitation from the congruent cues. It is not clear how any model which posits an independent segmentation process can explain this pattern; readers should use their stored knowledge about the characters that likely mark the beginnings or ends of words to segment the text into word units, thus allowing access to word-level representations, from which top-down activation is not available until the initial attempt at segmentation is complete. It is not clear how any such model could explain the lack of a benefit to reading times on the congruent pseudowords.

In contrast, the holistic hypothesis proposes a single-stage process that incorporates both segmentation and identification, and that receives top-down activation from word-level representations. If this were correct then controlling neighborhood size across the three conditions should have resulted in no significant differences in reading times, as all types of pseudoword should have received a matched degree of top-down activation from the orthographic neighbors. Despite the apparent failure to meet the predictions, we argue that our data are most consistent with such a single-stage model as per Li et al [3], as there are two key aspects of our data that warrant further consideration: (a) there was a significant cost associated with the incongruent pseudowords; and (b) when overall neighborhood size was controlled, this cost occurred in the learning phase but was not maintained at test (in contrast to Experiment 1, where orthographic neighbors were not controlled).

The first of these points relates to the question of why, when overall neighborhood size was controlled, did we continue to observe a cost to processing for incongruent pseudowords, but no facilitation for congruent pseudowords? It is important to note that top-down activation can be both consistent with and contradictory to the orthographic input. Consistent with the published literature on Chinese reading [46], we calculated neighborhood size as being those 2-character words that shared one of the two constituent characters in its specified position (e.g., C1 shared the first position and the identity of C2 varying, or C2 shared in the second position and the identity of C1 varying); we refer to these as position-matched neighbors. The word-level representations of these position-matched neighbors should provide facilitatory, top-down activation to both segmentation and identification based on their partial but consistent overlap with the orthographic input. Critically, however, there will be other words that have the same (50%) overlap for the identity of the constituent characters but that are mismatched in terms of the location of the shared character (e.g., C1 shared but in the second position, with the identity of C1 varying, or C2 shared but in the first position, with the identity of C2 varying); we refer to these as position-mismatched neighbors. The word-level representations from these position-mismatched neighbors could be inhibitory, as that feedback would contain incorrect, misleading information about the locations of word boundaries. In such a way, we suggest that top-down feedback from position-mismatched word neighbors may underlie the patterns in our data. We calculated the number of position-mismatched neighbors for the stimuli in each of our three conditions, the results showed that the position-mismatched neighborhood sizes for both congruent and balanced pseudowords were significantly smaller than those for incongruent pseudowords (*t*s > 4, both *p*s < 0.001). This supports our argument that a dominant proportion of the top-down activation for the incongruent pseudowords came from lexical entries that partially matched the identity of the orthographic input but that contradicted the positional information (these latter cues being indicative of the likely locations of word boundaries). To reiterate, this pattern of results that is associated with top-down activation from word-level representations cannot be explained by a model that assumes lexical segmentation to be separate from, and to precede, lexical identification. Rather, these data support a holistic model, such as that proposed by Li et al [3], in which Chinese lexical segmentation and identification are part of the same process.

Finally, we consider the second aspect of our data which speaks to this point—the finding that the cost to processing associated with incongruent pseudowords in the learning phase was not maintained at test when the number of position-matched neighbors was controlled. As this point requires a comparison of the data from both Experiments 1 and 2, we move to the *General Discussion*.

## General discussion

In these experiments, a pseudoword learning paradigm was used to examine how Chinese readers use characters’ positional frequencies to identify novel words during sentence reading, when presented with/ without word spacing. We found that our manipulation of character positional frequency did affect reading times on the target pseudowords but, critically, we found little evidence of an interaction with our manipulation of word spacing. This suggests that processing of the constituent characters’ positional frequencies and of word spacing are independent. We will consider the implications of these two independent effects in turn.

### Character positional frequency effect on word learning

Note that, across both experiments, there was no evidence whatsoever for a processing advantage for new words that contained strong, and correct, character positional frequency cues (relative to the balanced condition, which did not contain any useful cues for word boundaries). All effects of character positional frequencies upon the efficiency of word learning were driven by a cost to processing incongruent pseudowords. We argued that the cost to processing that was observed for reading times on the incongruent pseudowords in Experiment 2 was most likely associated with the high number of position-mismatched neighbors. An outstanding question is, then, why was a change in the maintenance of this effect between learning and test phases observed across the two experiments? Once again, a likely explanation comes from the influence of top-down activation from word-level representations, and so we focus on the data from Experiment 2 where this was controlled (in contrast to Experiment 1, where it was not). Top-down activation from orthographic neighbors would have played a stronger role in the learning phase—at that point, the reader did not have a lexical representation that fully matched the orthographic input and so top-down activation would have been limited to neighbors that only partially matched the input. Consistent with this suggestion, in Experiment 2, it was in the learning phase that we observed a significant cost to processing incongruent pseudowords. As discussed previously, this was likely the result of a high number of position-mismatched neighbors providing incorrect feedback about word boundary locations within the stimuli. In the subsequent test phase, participants would have formed new lexical representations for the pseudowords, albeit not fully specified; it is, therefore, perhaps unsurprising that the cost for incongruent pseudowords was not maintained at test—the influence of position-mismatched neighbors would have been attenuated by the presence of a lexical representation that fully matched the orthographic input.

A final, outstanding question about the effects of the positional frequency manipulation across the two experiments is: why did the time course of the effect change within the eye movement behaviour? In Experiment 1, there was a relatively early cost to processing incongruent pseudowords (appearing in first fixation durations). In Experiment 2, the effect was delayed in terms of the eye movement measures (occurring only in total fixation time but not in first-pass reading times). The most likely explanation for this is the readers’ reliance upon the sentence context for corrections to errors in lexical segmentation, which is subsequent to initial processing of cues from word-level representations. Again, therefore, we focus upon the data from Experiment 2 where neighborhood size was controlled. It seems possible that, in the case of the incongruent pseudoword, the reader’s initial attempt at segmentation/identification of a given stimulus based on the characters’ positional frequencies might be incorrect. Once the reader discovers that the initial segmentation is inconsistent with the sentential context, then there should be some secondary, corrective attempt at segmentation. It would seem likely that such a pattern of an initial segmentation error followed by a correction on the basis of the sentence context might be more common for the incongruent pseudowords than for the balanced or congruent pseudowords.

The data from these two experiments provide some clear conclusions with respect to character positional frequency cues. First, when processing new vocabulary within a sentence, readers are sensitive to the positional frequencies of the constituent characters, and this is independent from orthographic neighborhood size. Second, these effects are likely to be a consequence of activation from lexical representations that partially match the identity, but contradict the positional information, of the constituent characters. Third, and critically, the attribution of these effects to top-down activation from word-level representations provides strong evidence in support of a holistic hypothesis of Chinese lexical segmentation and identification, as per [3]. It is not clear how any model that proposes a lexical segmentation process that precedes lexical identification could explain the current pattern of results.

### Word spacing effect on word learning

The highly consistent result between the two experiments was that readers in the unspaced learning group had a cost to their reading times on pseudowords in the learning phase, but readers in the spaced learning group did not. This suggests that the presence of word spacing allowed Chinese readers to learn these new words as efficiently as possible during the initial encounters within sentence contexts, such that even the subsequent removal of word spacing during the test phase was not detrimental. Reading new words in a spaced format for the first few encounters facilitated learning of those words, and that learning was independent of the format on the printed page.

As stated by Liang et al (2015), when a reader encounters a novel word that is embedded in traditional, unspaced text, there are no salient visual segmentation cues available for the reader to determine which character(s) might comprise the new word. Recall that the majority of Chinese characters do not uniquely appear at the beginnings or ends of words; any given character could be a single-character word, or occur in the beginning, middle or end position of one of a number of different multi-character words [3, 6]. In an unspaced format, therefore, readers have to depend on the linguistic content of the text (character cues, lexical cues, and the sentence context) to determine how many and which adjacent characters make up the novel word. Only once the correct word segmentation has occurred, it is possible for the reader to confirm a mismatch between the current orthographic input and any existing representation in the mental lexicon and, consequently, to initiate the formation of a new orthographic representation. In contrast, when reading word-spaced text, it seems likely that the presence of word spacing provides the reader with a clear (and correct) visual cue as to how many and which adjacent characters comprise each word unit within the sentence. On the basis of this information the reader can determine, for each unit, whether it is a word already known to them or whether it is an unknown word. Thus, spacing facilitates the instantiation of new lexical entries.

### Summary and conclusions

The present experiments showed that the positional frequencies of a word’s constituent characters influenced Chinese lexical acquisition regardless of the presence or absence of word spacing. In terms of the role of word spacing on Chinese lexical acquisition—it allowed Chinese readers to quickly and accurately determine the presence of an unknown word, and to instantiate a new lexical representation by providing visual demarcation of word boundaries. It was likely that the positional frequencies of a word’s constituent characters influenced the character-to-word assignment, in a process that likely incorporates both lexical segmentation and identification. It is important that current models of Chinese lexical identification in reading are extended to provide some account of the lexical acquisition process. The present data set, along with other recent experimental work in this area [1, 10] provide important evidence both of critical characteristics of written language that impact upon the process of learning new words (word spacing, and the positional frequencies of constituent characters), and how these characteristics are influential during reading.

## Supporting information

S1 Table Data from Experiment 1. (XLS)

S2 Table Data from Experiment 2. (XLS)

S3 Table Stimuli from Experiments 1 & 2. (XLSX)
